# Effect of long-term fertilization strategies on bacterial community composition in a 35-year field experiment of Chinese Mollisols

**DOI:** 10.1186/s13568-018-0549-8

**Published:** 2018-02-13

**Authors:** Mingchao Ma, Jing Zhou, Marc Ongena, Wenzheng Liu, Dan Wei, Baisuo Zhao, Dawei Guan, Xin Jiang, Jun Li

**Affiliations:** 1 Institute of Agricultural Resources and Regional Planning, Chinese Academy of Agricultural Sciences, Beijing, 100081 China; 2 Microbial Processes and Interactions Research Unit, Gembloux Agro-Bio Tech, University of Liège, Passage des Déportés, 2, Gembloux, Belgium; 3 Laboratory of Quality & Safety Risk Assessment for Microbial Products, Ministry of Agriculture, Beijing, 100081 China; 4 The Institute of Soil Fertility and Environmental Sources, Heilongjiang Academy of Agricultural Sciences, Harbin, 150086 People’s Republic of China

**Keywords:** Soil degradation, Fertilization, Bacterial community, Illumina MiSeq, qPCR

## Abstract

Bacteria play vital roles in soil biological fertility; however, it remains poorly understood about their response to long-term fertilization in Chinese Mollisols, especially when organic manure is substituted for inorganic nitrogen (N) fertilizer. To broaden our knowledge, high-throughput pyrosequencing and quantitative PCR were used to explore the impacts of inorganic fertilizer and manure on bacterial community composition in a 35-year field experiment of Chinese Mollisols. Soils were collected from four treatments: no fertilizer (CK), inorganic phosphorus (P) and potassium (K) fertilizer (PK), inorganic P, K, and N fertilizer (NPK), and inorganic P and K fertilizer plus manure (MPK). All fertilization differently changed soil properties. Compared with CK, the PK and NPK treatments acidified soil by significantly decreasing soil pH from 6.48 to 5.53 and 6.16, respectively, while MPK application showed no significant differences of soil pH, indicating alleviation of soil acidification. Moreover, all fertilization significantly increased soil organic matter (OM) and soybean yields, with the highest observed under MPK regime. In addition, the community composition at each taxonomic level varied considerably among the fertilization strategies. Bacterial taxa, associated with plant growth promotion, OM accumulation, disease suppression, and increased soil enzyme activity, were overrepresented in the MPK regime, while they were present at low abundant levels under NPK treatment, i.e. phyla *Proteobacteria* and *Bacteroidetes*, class *Alphaproteobacteria*, and genera *Variovorax*, *Chthoniobacter*, *Massilia*, *Lysobacter*, *Catelliglobosispora* and *Steroidobacter*. The application of MPK shifted soil bacterial community composition towards a better status, and such shifts were primarily derived from changes in soil pH and OM.

## Introduction

Mollisols (black soils) are widely distributed in the northeast of China and are considered to be highly fecund and productive (Wei et al. 2008). However, these soil have degenerated over time due to intensive fertilizer application (Liu et al. 2015). The widespread use of inorganic fertilizers has also reduced black soil quality and overall environmental health since large scale reclamation was initiated around the middle of the last century (Yin et al. 2015). Take soil OM for example, the original level of soil OM content on the top layer (0–20 cm) is about 10% or more, as soil OM accumulates faster than it is decomposed during the relative cold seasons (Wen and Liang 2001). After farming started, soil OM content drastically dropped to half of its original level in 20–30 years, and now stabilized at 2–4%. In addition, overuse of N fertilizer has caused soil acidification, as well as a reduction in soil microbial biomass and bacterial diversity (Guo et al. 2010). Our previous studies found that long-term N application changed the microbial community composition in black soils by reducing bacterial and fungal diversity and the bacteria to fungi ratio, which led to soil degradation (Zhou et al. 2015, 2016; Ding et al. 2016). Constant N input can also lead to changes in plant species composition and a loss of plant diversity (Clark et al. 2007). In response to soil degradation associated with excessive inorganic N fertilizer, reductions in inorganic N input have been advocated.

In contrast to inorganic N fertilizers, the benefits of organic fertilizer for agricultural production and soil fertility cannot be overemphasized. Organic fertilizer can increase soil organic carbon and total N (TN) (He et al. 2015), improve soil aggregate stability (Xie et al. 2015) and have residual N effects in subsequent years (Schröder et al. 2007). Manure, an important source of organic matter, can improve disordered bacterial community structures in soil that result from the overuse of inorganic fertilizer (Ai et al. 2015), thereby improving soil quality (Davidson 2009). Several studies have explored the influence of fertilization on plant communities, soil properties, microbial community structure, and microbial activity (Hallin et al. 2009; Ramirez et al. 2010; Wertz et al. 2012; Hartmann et al. 2013; Zhong et al. 2015). However, little is known about the overall impacts of different fertilization strategies on bacterial community composition, especially when manure is substituted for inorganic N fertilizer. In this study, soils were collected from a 35-year field experiment of Chinese Mollisols, which was ideal for investigating the effects of fertilization strategies on soil microorganisms (Geisseler and Scow 2014). We used high-throughput pyrosequencing and qPCR technology to describe soil bacterial community composition under organic manure and inorganic fertilizer regimes. Here, we hypothesize that (1) bacterial community composition and abundance differ among fertilization strategies due to altered soil properties, especially soil pH and OM; and (2) manure shifts soil bacterial communities towards a better status, whereas inorganic fertilizer has the opposite effect. In summary, understanding the responses of bacterial community composition to different fertilization strategies is an effective way to reveal the relationship between intensive fertilization and black soil degradation. The results highlight the potential use of manure for sustainable development in Chinese Mollisols.

## Materials and methods

### Field experiments and soil sampling

The field experiment, established in 1980, was located at the Heilongjiang Academy of Agricultural Sciences, Heilongjiang Province, China (45°40′N, 126°35′E, altitude 151 m). The experiment was set up as a block design and each block was treated with different fertilizer strategies in triplicate: no fertilizer (CK), inorganic phosphorus (P) and potassium (K) fertilizer (PK), inorganic N, P and K fertilizer (NPK), and inorganic P and K fertilizer plus manure (MPK). Blocks were randomized into plots of 9 × 4 m. Doses of inorganic fertilizers were 75 kg ha^−1^ N (urea), 150 kg ha^−1^ P_2_O_5_, 75 kg ha^−1^ K_2_O and 18,600 kg ha^−1^ horse manure. Soils were collected from plant rows after soybean harvest in September 2014.

For each replicate plot, six cores were collected from the topsoil (5–20 cm) using a 3-cm diameter soil corer. Plant residue and gravel were removed and samples were mixed uniformly to form one composite sample. Each composite sample was divided into three parts. One part was stored at − 80 °C and the other two were used as two independent samples. Thus, a total of 24 soil samples were obtained for analyses.

### Soil properties and soybean yield

Prior to chemical characterization, soil samples were air dried at room temperature and passed through a 2.0 mm sieve. Soil samples were diluted 1:1 in water and pH was measured using a pH meter. Soil OM was measured using the K_2_Cr_2_O_7_-capacitance method (Strickland and Sollins 1987). The Kjeldahl method was used to measure TN (Huang et al. 2007). NH_4_^+^–N and NO_3_^−^–N were tested according to Hart et al. (1994). Atomic absorption spectrometer and flame photometry were used to measure total K (TK) and available K (AK) (Helmke and Sparks 1996). The total P (TP) and available P (AP) were determined by colorimetric methods (Garg and Kaushik 2005) and resin extraction with modification (Hedley and Stewart 1982), respectively. Soybean yields were recorded after harvest.

### High-throughput pyrosequencing and qPCR analysis

Total DNA was extracted using a MOBIO PowerSoil DNA Isolation Kit (Qiagen, Carlsbad, CA, USA) with modifications to the incubation step as previously described (Fierer et al. 2012b). For each of the 24 soil samples, six replicate extractions were combined together to obtain sufficient quantities of homogeneous DNA (Ding et al. 2016). DNA was purified, and then, DNA concentration and quality (A_260_/A_280_) of the extracts were estimated visually using a NanoDrop ND-1000 UVevis spectrophotometer (Thermo Scientific, Rockwood, TN, USA). The V4 region of the 16S rRNA gene was amplified using primers 515F and 806R (Peiffer et al. 2013), which were designed to be universal for bacterial and archaeal taxa (Ramirez et al. 2012). Given the rare abundance of archaea (normally less than 1% of sequences), only the results for bacterial communities are presented. Illumina MiSeq Sequencing was carried out at the Personal Biotechnology Co. Ltd. (Shanghai, China), according to the methods of Caporaso et al. (2012). The 16S rRNA gene sequences were submitted to the NCBI Sequence Read Archive with the Accession Number SRP 045472.

In spite of some inherent limitations, qPCR can be still used to estimate microbial abundance (Liu et al. 2015). The 515F/806R primer set was used for qPCR using an Applied Biosystems 7500 detection system (Applied Biosystems, Foster City, CA, USA). The reaction mixture (25 μL) and amplification conditions were performed according to the methods of Lauber et al. (2013) and Zhou et al. (2015). The qPCR was carried out in triplicate for each extracted DNA sample.

### Bioinformatics and statistical analyses

Mothur software (v1.32, http://www.mothur.org/) was used to assemble pyrosequencing reads as described by Schloss et al. (2011). Operational taxonomic units (OTUs) were identified using a cut-off of 97% similarity and were invalid in the case that less than four replicates were detected in one sample. Singletons, non-bacterial OTUs were removed, and the OTU abundance levels were normalized based on the sample with the least number of sequences. To perform a fair comparison between samples, all subsequent analyses were performed according to the normalized data (Zhou et al. 2016). The Ribosomal Database Project Naïve Bayesian rRNA classifier was used with a minimum percent identity threshold of 60% for taxonomic assignment (Li et al. 2014). Bacterial α-diversity (CHAO1, ACE and Shannon and Simpson indices) was calculated with ten times subsampling using Mothur software (v1.32). Weighted UniFrac distances were calculated and principal coordinates analysis (PCoA) was carried out to identify variations in bacterial community composition. Linear discriminant analysis coupled with effect size (LEfSe) was performed to identify significant differences in abundance of bacterial genera between MPK and NPK treatments (Segata et al. 2011). The linear discriminant analysis (LDA) score threshold was set to greater than 3.0. Relationships between bacterial community composition and soil properties were revealed by redundancy analysis (RDA) using CANOCO 5.0 software. Variance analysis of all experimental data was performed using SPSS (v.19). In all tests, *P* < 0.05 were considered statistically significant.

## Results

### Soil properties and soybean yields

The three fertilization strategies significantly increased concentrations of AP, AK, TP and TK (Table 1). Compared with CK, both NPK and MPK treatments increased the concentrations of NO_3_^−^ and TN. NPK and PK significantly decreased soil pH from 6.48 to 5.53 and 6.16, respectively, while the MPK application alleviated soil acidification. Moreover, MPK treatment also had an accumulative effect on soil OM. In addition, soybean yields were significantly higher under the fertilization regimes, with the MPK application being the most effective strategy (2702 kg ha^−1^).

**Table 1:** Soil properties and soybean yields of different fertilization strategies

Fertilization strategy	pH	OM (g kg^−1^)	AK (g kg^−1^)	TK (g kg^−1^)	NH_4_^+^ (mg kg^−1^)	NO_3_^−^ (mg kg^−1^)	TN (g kg^−1^)	AP (g kg^−1^)	TP (g kg^−1^)	Soybean yield (kg ha^−1^)
CK	6.43 ± 0.08c	24.39 ± 0.37a	0.17 ± 0.03a	6.30 ± 0.89a	35.01 ± 1.16a	2.45 ± 0.88a	1.20 ± 0.05a	0.02 ± 0.01a	0.44 ± 0.03a	1812.67 ± 141.99a
PK	6.18 ± 0.04b	25.51 ± 0.30c	0.24 ± 0.01b	28.57 ± 2.25b	37.80 ± 2.95a	3.62 ± 0.57b	1.26 ± 0.03a	0.89 ± 0.04c	0.73 ± 0.02c	2377.33 ± 118.85bc
NPK	5.54 ± 0.04a	24.88 ± 0.25b	0.23 ± 0.03b	30.36 ± 1.02b	37.30 ± 6.29a	4.53 ± 0.91bc	1.43 ± 0.08b	0.94 ± 0.06d	0.70 ± 0.02bc	2241.33 ± 186.11b
MPK	6.38 ± 0.05c	27.47 ± 0.41d	0.23 ± 0.03b	28.43 ± 3.93b	39.36 ± 6.95a	5.17 ± 0.67c	1.20 ± 0.03a	0.66 ± 0.01b	0.61 ± 0.05b	2702.67 ± 169.39c

Values are mean ± standard deviation (n = 6). Values within the same column followed by different lowercase letters indicate significant differences according to Tukey’s multiple comparison tests

## 16S rRNA gene abundances

**Fig. 1:**
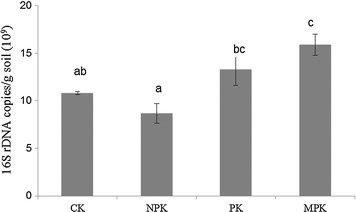
Number of 16S rDNA copies in different fertilization regimes. Different lowercase letters above columns indicate significant differences according to Tukey’s multiple comparison tests

The effect of different fertilization strategies on 16S rRNA gene abundances was assessed. The number of gene abundances ranged from 8.69 × 10^9^ to 1.59 × 10^10^ g^−1^ soil (Fig. 1). PK and MPK treatments significantly increased, whereas NPK treatment decreased the number of 16S rRNA gene abundances compared with CK.

### Bacterial α-diversity

A total of 538,485 high-quality sequences (70% of total sequences) were detected with an average read length of 270 bp. Based on a similarity cut-off of 97%, the minimum Good’s coverage value was 0.95, meaning that a sufficient number of reads were obtained to evaluate bacterial diversity. With regards to bacterial α-diversity (Table 2), NPK treatment significantly decreased CHAO1 and the Shannon indices in comparison with the other three treatments.

**Table 2:** Bacterial α-diversity and Good’s coverage estimator for different fertilization strategies

Fertilization strategy	CHAO1	Ace	Simpson	Shannon	Coverage
CK	3231.9 ± 133.0b	3347.9 ± 149.0a	0.0076 ± 0.0007a	6.3 ± 0.03b	0.95 ± 0.006a
PK	3262.7 ± 154.8b	3386.1 ± 256.1a	0.0076 ± 0.0012a	6.3 ± 0.05b	0.96 ± 0.007a
NPK	2866.6 ± 63.3a	3328.8 ± 414.7a	0.0115 ± 0.0009b	6.0 ± 0.02a	0.95 ± 0.006a
MPK	3421.6 ± 94.3b	3541.9 ± 98.8a	0.0077 ± 0.0005a	6.3 ± 0.03b	0.96 ± 0.001a

Values are mean ± standard deviation (n = 6). Values within the same column followed by different lowercase letters indicate significant differences according to Tukey’s multiple comparison tests. Operational taxonomic units defined based on a 97% similarity threshold

### Bacterial community composition

Relative abundances at the phylum level (> 1%) are shown in Table 3. All samples were dominated by the phyla *Proteobacteria*, which accounted for 29.59–35.73% of the total sequences, followed by *Acidobacteria* (13.23–16.39%), *Actinobacteria* (9.26–10.83%), *Verrucomicrobia* (8.62–9.92%), and *Planctomycetes* (7.03–8.04%). MPK treatment resulted in the highest abundance of *Proteobacteria*. Moreover, the abundance of *Bacteroidetes* and *Nitrospirae* were also higher in MPK regime than those of NPK.

**Table 3:** Relative abundance of bacterial phyla of different fertilization strategies (relative abundance > 1%)

Phylum	CK (%)	PK (%)	NPK (%)	MPK (%)
*Proteobacteria*	30.79 ± 0.98ab	29.59 ± 1.00a	33.47 ± 0.57ab	35.73 ± 8.96b
*Acidobacteria*	15.92 ± 0.94b	16.39 ± 1.78b	13.61 ± 1.26a	13.23 ± 1.56a
*Actinobacteria*	10.30 ± 0.83a	9.35 ± 3.54a	10.83 ± 1.46a	9.26 ± 3.29a
*Verrucomicrobia*	8.69 ± 0.33a	9.22 ± 1.57a	9.92 ± 0.84a	8.62 ± 1.32a
*Planctomycetes*	7.32 ± 0.36ab	8.04 ± 0.74b	7.66 ± 0.52ab	7.03 ± 0.96a
*Gemmatimonadetes*	7.23 ± 1.07ab	7.24 ± 0.92a	8.40 ± 0.91b	6.03 ± 1.11ab
*Chloroflexi*	7.51 ± 0.28b	6.94 ± 0.6ab	6.50 ± 0.47a	6.49 ± 1.34a
*Bacteroidetes*	4.18 ± 1.36ab	4.82 ± 1.49ab	3.04 ± 0.37a	5.35 ± 1.8b
*Thaumarchaeota*	2.89 ± 0.69a	3.47 ± 1.59a	3.62 ± 0.70a	3.76 ± 1.61a
*Nitrospirae*	2.71 ± 0.28ab	2.49 ± 0.18ab	1.01 ± 0.12a	2.40 ± 0.30b

Values are mean ± standard deviation (n = 6). Values within the same column followed by different lowercase letters indicate significant differences according to Tukey’s multiple comparison tests

**Fig. 2:**
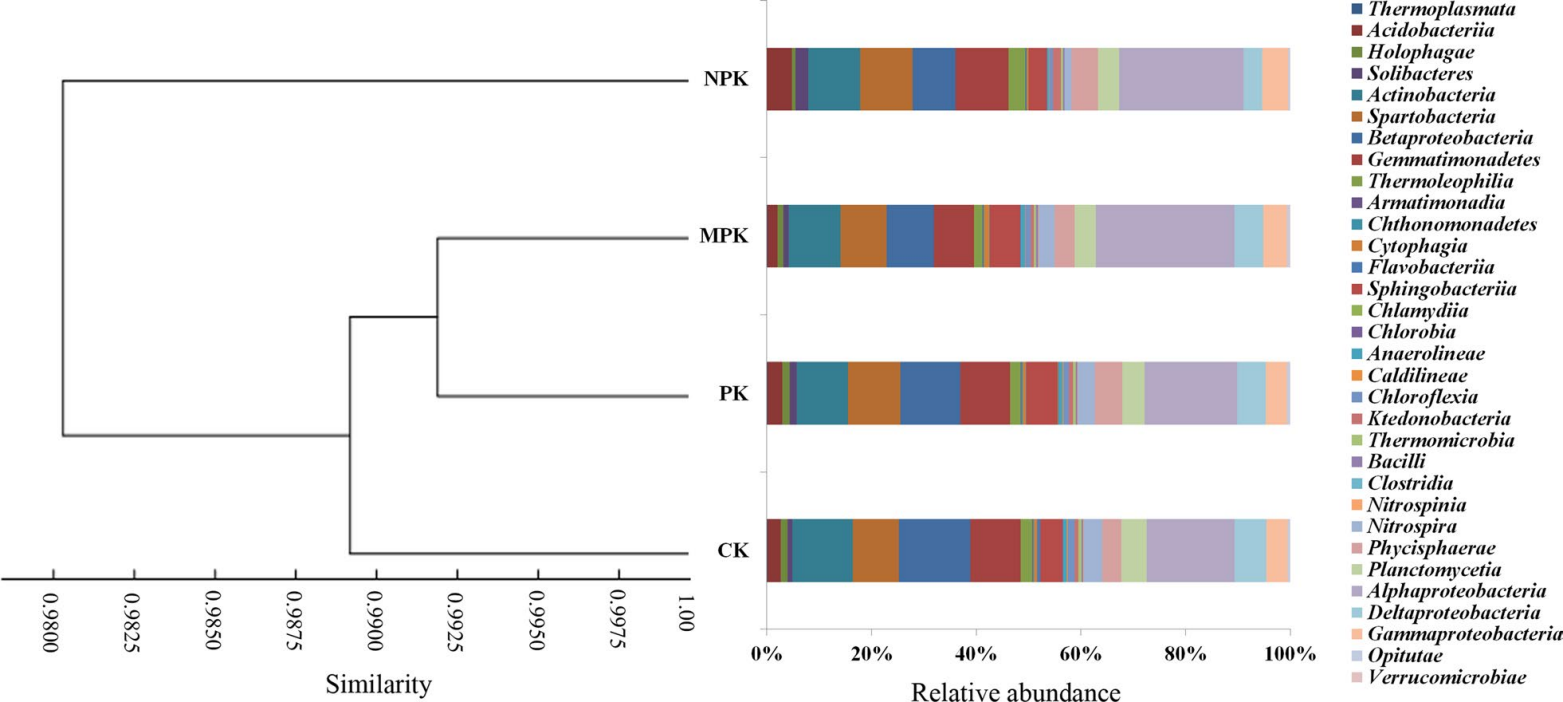
Cluster analysis of the 16S rDNA composition of soil-dwelling microbial communities at the class level

A total of 14 abundant classes (relative abundance > 1%) were identified (Table 4). *Alphaproteobacteria*, which represented 12.66–20.61% of the total sequences, was most abundant followed by *Betaproteobacteria* (6.74–10.48%), *Actinobacteria* (7.76–8.61%), *Spartobacteria* (6.75–8.36%) and *Gemmatimonadetes* (6.03–8.40%). NPK and MPK treatments significantly increased the relative abundance of *Alphaproteobacteria* and *Gammaproteobacteria*. *Betaproteobacteria* abundance was lower in the three fertilization strategies compared with CK, with the lowest abundance observed in NPK. NPK application also caused a significant reduction in the abundance of *Deltaproteobacteria*. Compared with CK, NPK application increased abundance of *Solibacteres*, *Thermoleophilia*, *Phycisphaerae*, *Acidobacteriia*, and *Gemmatimonadetes*, whereas these classes were present at low levels in MPK treated samples. NPK treatment resulted in the lowest abundance of *Nitrospira*, whereas abundance was significantly increased in the MPK regime. Cluster analysis demonstrated that bacterial community composition of PK, MPK and CK treatments clustered into one group with a similarity of 98.85% that was separated from the NPK treatment (Fig. 2).

**Table 4:** Relative abundance of bacterial classes of different fertilization strategies (relative abundance > 1%)

Phylum	Class	CK (%)	PK (%)	NPK (%)	MPK (%)
*Proteobacteria*	*Alphaproteobacteria*	12.66 ± 0.88 a	13.39 ± 0.84a	19.72 ± 0.77b	20.61 ± 10.18b
	*Betaproteobacteria*	10.48 ± 0.97c	8.86 ± 1.58b	6.74 ± 0.51a	7.13 ± 0.81a
	*Deltaproteobacteria*	4.53 ± 0.57b	4.17 ± 0.37b	2.81 ± 0.17a	4.32 ± 0.32b
	*Gammaproteobacteria*	3.06 ± 0.49a	3.09 ± 0.46a	4.04 ± 0.38b	3.59 ± 0.18b
*Acidobacteria*	*Acidobacteriia*	1.90 ± 0.13b	2.18 ± 0.20b	3.88 ± 0.45c	1.51 ± 0.17a
	*Solibacteres*	0.62 ± 0.14a	1.00 ± 0.21b	2.02 ± 0.15c	0.78 ± 0.11a
*Actinobacteria*	*Actinobacteria*	*8.61* *±* *0.58a*	7.76 ± 2.99a	8.19 ± 1.10a	7.89 ± 2.81a
	*Thermoleophilia*	*1.69* *±* *0.27a*	1.59 ± 0.56a	2.65 ± 0.40b	1.37 ± 0.49a
*Verrucomicrobia*	*Spartobacteria*	6.75 ± 0.52a	7.47 ± 1.47ab	8.36 ± 0.98b	7.06 ± 1.25ab
*Planctomycetes*	*Phycisphaerae*	2.77 ± 0.15a	3.97 ± 0.41b	4.20 ± 0.42b	3.05 ± 0.52a
	*Planctomycetia*	3.52 ± 0.29a	3.27 ± 0.25a	3.19 ± 0.18a	3.13 ± 0.48a
*Gemmatimonadetes*	*Gemmatimonadetes*	7.23 ± 1.07ab	7.24 ± 0.92ab	8.40 ± 0.91b	6.03 ± 1.11a
*Bacteroidetes*	*Sphingobacteriia*	3.17 ± 0.73ab	4.25 ± 1.32b	2.75 ± 0.30a	4.45 ± 1.55b
*Nitrospirae*	*Nitrospira*	2.71 ± 0.28c	2.49 ± 0.18bc	1.01 ± 0.12a	2.40 ± 0.30b

Values are mean ± standard deviation (n = 6). Values within the same column followed by different lowercase letters indicate significant differences according to Tukey’s multiple comparison tests

**Fig. 3:**
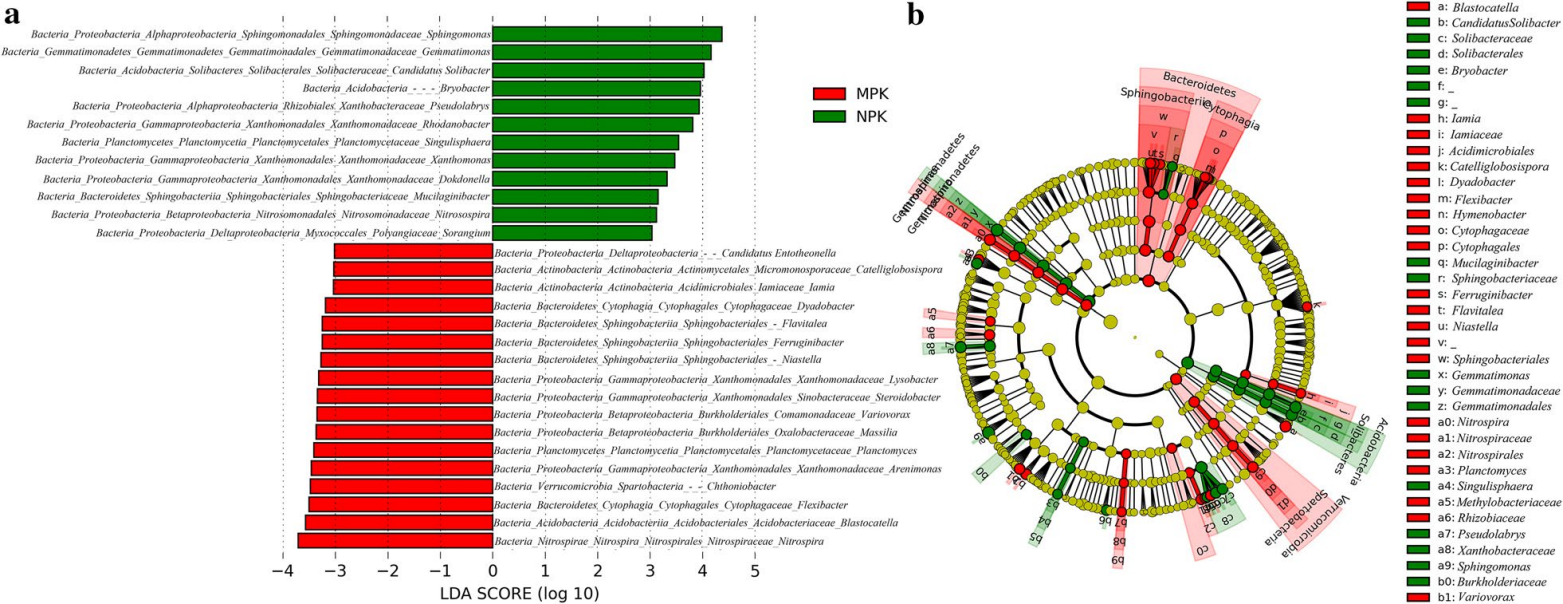
Bacterial taxa with significantly different abundances between NPK and MPK treatments. **a** Histogram of LDA scores for features with significantly different abundance between NPK and MPK treatments. **b** Taxonomic representation of statistically and biologically consistent differences between NPK and MPK treatments

At the genus level, significantly different taxa (average relative abundance > 0.01%) were identified between NPK and MPK regimes (Fig. 3). Many bacterial genera were significantly more abundant in the NPK regime (e.g. *Sphingomonas*, *Gemmatimonas*, *Bryobacter*, *Rhodanobacter*, *Xanthomonas*, *Nitrosospira*, *Mucilaginibacter*, etc.), whereas other taxa were overrepresented in the MPK regime (e.g. *Nitrospira*, *Blastocatella*, *Flexibacter*, *Chthoniobacter*, *Catelliglobosispora*, *Massilia*, *Variovorax*, *Steroidobacter*, *Lysobacter*, etc.).

### Bacterial β-diversity

**Fig. 4:**
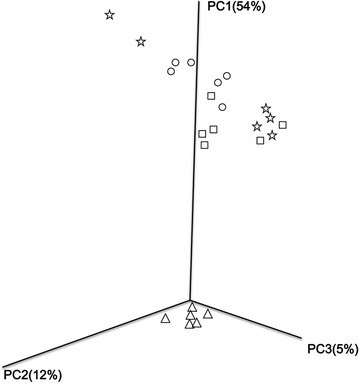
Principal components analysis of pyrosequencing reads obtained from soils treated with different fertilization strategies based on the weighted Fast UniFrac metric. The first three axes are drawn and the percent of variance explained by each axis is given. Treatment: (circle) CK, no fertilizer; (square) PK, inorganic phosphorus and potassium fertilizer; (triangle) NPK, inorganic P, K and N fertilizer; (star) MPK, inorganic P and K fertilizer plus manure

The results of PCoA analysis based on the weighted UniFrac distance matrix showed a clear separation between different fertilization strategies (Fig. 4). PC1, PC2 and PC3 explained 54, 12 and 5% of the variation, respectively. CK, PK and MPK plots were clustered together in the upper part, whereas the NPK plot was located separately in the bottom.

### Relationship between bacterial community structure and soil properties

**Fig. 5:**
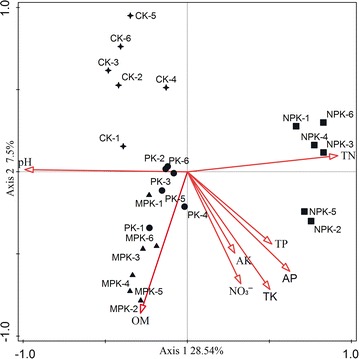
Redundancy analysis (RDA) of soil bacterial composition and soil properties. Soil factors indicated in arrows include Avail P (available phosphorus), Avail K (available potassium), pH, NO3– (nitrate nitrogen), TN (total nitrogen), TK (total potassium), TP (total phosphorus) and OM (organic matter). Treatment: (star) CK, no fertilizer; (circle) PK, inorganic phosphorus and potassium fertilizer; (square) NPK, inorganic P, K and nitrogen fertilizer; (triangle) MPK, inorganic P and K fertilizer plus manure

Based on RDA analysis, selected soil properties (soil pH, AP, AK, NH_4_^+^, NO_3_^−^, TK, TP, TN and OM) accounted for 54.2% of the variance of the model (Fig. 5). The first and second axes explained 28.54 and 7.5% of the total variation, respectively. Compared with NPK, CK, PK, and MPK treatments clustered together in the first and fourth quadrants, confirming the results of the cluster analysis. The selected soil properties affected the bacterial community composition in the following order: pH > OM > TN > AP > AK > TP > NO_3_^−^ > TK.

## Discussion

### Inorganic PK plus manure application increased soybean yield and improved soil quality

Although all tested fertilization treatments increased soybean yield, MPK treatment was considered to be the optimal fertilization strategy, which were attributed to the slow release of manure nutrients into the soil (Zhao et al. 2014). PK treatment also resulted in higher soybean yield than NPK treatment, indicating a negative effect of inorganic N fertilizer. Indeed, high available N prevents plants from providing carbon for nutrient-absorbing systems (Wei et al. 2013). Inorganic N has also been shown to inhibit biological N fixation, which provides N needed for soybean growth (Gelfand and Robertson 2015).

In agreement with previous findings that the application of inorganic fertilizer alone can acidify soil (van Diepeningen et al. 2006), we found that both NPK and PK treatments significantly decreased soil pH. In particular, a decline of almost one pH unit was detected in response to NPK treatment in our study. In analysis and comparison of 10 long-term experimental fields, Guo et al. (2010) also found that significant soil acidification occurs in NPK-treated plots (*P* < 0.001). In contrast, MPK application had positive effects on the alleviation of soil acidification due to the buffering function of carbonates, bicarbonates, carboxyl and phenolic hydroxyl groups (Whalen et al. 2000; Garcia-Gil et al. 2004). Soil OM also accumulated in response to MPK treatment, which might be attributed to the macronutrient status of manure (Xie et al. 2014). Consequently, manure might stimulate the microbial biomass and increase soybean yield. With increased productivity, the amount of soybean residues returned to the soil after harvest also increases, leading to increased OM as residues decompose over time (Geisseler and Scow 2014). Overall, manure may be a potential substitute for inorganic N fertilizers for improvement of soybean growth and soil quality.

### Inorganic PK plus manure application increased bacterial diversity

The number of 16S rRNA gene abundances was significantly different between the fertilization strategies. Compared with CK, MPK treatment significantly increased, whereas NPK treatment decreased the number of 16S rRNA gene abundances. Soil pH has been identified as a crucial factor in determining bacterial population dynamics (Zhou et al. 2015), since the optimal pH range for bacterial growth is limited (Rousk et al. 2010a). Ahn et al. (2012) also reported that bacterial abundance was correlated with soil pH.

There were no significant differences in CHAO1 and Shannon indices between CK, PK, and MPK regimes. However, similar to previous reports (Geisseler and Scow 2014; Zeng et al. 2016), NPK treatment significantly decreased these indices, indicating that inorganic N fertilizer has a greater influence than P or K on ecosystem instability (Chaer et al. 2009). Organic manure has been shown to have profound effects on bacterial population diversity (Naeem and Li 1997) and nutrient cycling rates (Philippot et al. 2013), which play an important role in microbial functions and processes (Chaer et al. 2009).

### Inorganic PK plus manure application improved bacterial community composition

In the current study, *Proteobacteria* was the dominant phylum in all samples, which may be explained by the fact that members of this phylum can utilize a wide range of complex organic molecules and survive in various habitats (Bouskill et al. 2010). MPK treatment had the highest abundance of *Proteobacteria*, which were attributed to the greatest amount of soil nutrients available for copiotrophic bacterial growth (Fierer et al. 2012a). High abundance of *Proteobacteria* is particularly important for soybean growth, as members of this phylum have been shown to promote plant growth and facilitate horizontal transfer of genes related to photosynthesis (Makhalanyane et al. 2015). Additionally, many taxa within *Proteobacteria* had disease-suppression activity improving soil health (Mendes et al. 2011). *Acidobacteria* formed the second largest group in our dataset and was significantly lower in NPK and MPK regimes, which supported previous findings that oligotrophic bacteria such as *Acidobacteria* were negatively correlated with soil nutrients (Fierer et al. 2012a). Furthermore, MPK treatment significantly increased *Bacteroidetes* and *Nitrospirae* compared with NPK, indicating a positive effect on OM accumulation (Eilers et al. 2012) and nitrite oxidation (Cebron and Garnier 2005).

At the class level, bacterial community composition differed significantly among the fertilization treatments. Both NPK and MPK treatments significantly increased the abundance of *Alphaproteobacteria* and *Gammaproteobacteria*, probably due to the highly nutritious soil (Ahn et al. 2012). Increased abundance of these classes may be beneficial for soil quality, as *Alphaproteobacteria* can use recalcitrant forms of carbon and supply carbon intermediates to other microorganisms (Campbell et al. 2010). Additionally, members of the class *Gammaproteobacteria* have been shown to defend plants from fungal disease by a putative chlorinated lipopeptide (Mendes et al. 2011). NPK application led to a higher abundance of *Phycisphaerae* than CK and MPK treatments, probably increasing the performance of nitrate removal (Xiao et al. 2015). In addition, higher abundances of *Solibacteres* and *Thermoleophilia* were detected in response to NPK treatment, which confirmed previous findings that these two classes are positively correlated with N addition (Zhou et al. 2017). Finally, with the decline of soil pH, *Acidobacteriia* abundance increased in the NPK regime, suggesting that soil environment was favorable to acidophilic, chemoorganotrophic bacteria (Wu et al. 2017b).

LEfSe analysis was performed to identify significantly different genera between NPK and MPK treatments. Genera with negative impacts on soil quality were overrepresented in the NPK regime, i.e. *Sphingomonas*, *Xanthomonas*, *Rhodanobacter* and *Nitrosospira*, while they were present at low levels in the MPK treatment group. Multiple species of *Sphingomonas* and *Xanthomonas* are considered animal and plant pathogens (White et al. 1996; Barak et al. 2016). *Rhodanobacter* is likely involved in the denitrifying process leading to N losses in low pH soil (Green et al. 2012). Furthermore, besides ammonia oxidation, members in *Nitrosospira* can perform nitrified denitrification, resulting in reduced conversion of nitrite to N_2_O and emission of greenhouse gas (Shaw et al. 2006). In addition, MPK treatment increased the abundance of beneficial genera, such as *Variovorax*, *Chthoniobacter*, *Massilia*, *Lysobacter*, *Catelliglobosispora* and *Steroidobacter*. *Variovorax* is considered to be a plant growth-promoting rhizobacterium (Jiang et al. 2012) and *Chthoniobacter* plays important roles in carbohydrate metabolism (Brewer et al. 2016). Species in *Massilia* and *Lysobacter* produce violacein (Myeong et al. 2016) and lytic enzymes (Li et al. 2008) that allow them to colonize plant roots and protect against infection by soil-borne plant pathogens (Ko et al. 2009; Ofek et al. 2012). Similarly, higher abundances of *Catelliglobosispora* and *Steroidobacter* in the MPK treatment group may indicate improved soil saccharase (Sun et al. 2014) and catalase activity (Sakai et al. 2014), respectively. However, *Blastocatella* and *Nitrospira* were also increased, suggesting an increase in ammonia oxidation (Alma et al. 2016) and nitrite oxidation through the nitrification process (Wu et al. 2016). Wu et al. (2017a) explored the effects of fertilizer application over a 20-year period on soil N transformation and found that inorganic fertilizer plus manure increased N mineralization rate and available soil N. However, in practical applications it should be considered that higher N nitrification is also induced by manure application, which may lead to increased N losses (Wu et al. 2017a).

### Primary soil properties shape bacterial community composition

Soil microorganisms rapidly respond to changes in soil properties and these shifts can affect soil quality and plant growth (Marschner et al. 2003). In agreement with previous findings (Rousk et al. 2010b; Williams et al. 2013; Zhou et al. 2015), bacterial community composition was affected by soil pH and OM changes induced by long-term fertilization. Based on cluster and RDA analysis, the bacterial community composition of CK, PK and MPK treatment groups clustered together and were separated from NPK. Thus, inorganic N fertilizer input altered bacterial β-diversity. As Zhou et al. (2017) previously reported, long-term N application decreased soil pH and soil pH was highly correlated with UniFrac distance between bacterial communities. Soil pH plays a key role in shaping bacterial composition due the narrow pH range tolerated by most bacteria (Rousk et al. 2010a). Additionally, soil pH may indirectly affect bacterial community structure by responding to other variables and may provide an integrated index of soil conditions (Lauber et al. 2009). Hydrogen ion concentration varies by many orders of magnitude across various soils and, as numerous soil properties are related to soil pH, these factors may have driven the observed shifts in community composition (Xiong et al. 2012; Shen et al. 2013).

## Abbreviations

N: nitrogen
P: phosphorus
K: potassium
CK: no fertilizer
PK: inorganic phosphorus and potassium fertilizer
NPK: inorganic phosphorus, potassium and nitrogen fertilizer
MPK: inorganic phosphorus and potassium fertilizer plus manure
OM: organic matter
TN: total nitrogen
TK: total potassium
AK: available potassium
TP: total phosphorus
AP: available phosphorus
OTUs: operational taxonomic units
PCoA: principal coordinates analysis
LEfSe: linear discriminant analysis coupled with effect size
LDA: the linear discriminant analysis
RDA: redundancy analysis
